# Development and therapeutic manipulation of the head and neck cancer tumor environment to improve clinical outcomes

**DOI:** 10.3389/froh.2022.902160

**Published:** 2022-07-22

**Authors:** Thomas Duhen, Michael J. Gough, Rom S. Leidner, Sasha E. Stanton

**Affiliations:** Earle A. Chiles Research Institute, Providence Cancer Institute, Portland, OR, United States

**Keywords:** OSCC (oral squamous cell carcinoma), HNSCC (head and neck squamous cell carcinoma), CD8, CD4, pre-malignancies, TIL (tumor infiltrating lymphocytes), immunotherapy

## Abstract

The clinical response to cancer therapies involves the complex interplay between the systemic, tumoral, and stromal immune response as well as the direct impact of treatments on cancer cells. Each individual's immunological and cancer histories are different, and their carcinogen exposures may differ. This means that even though two patients with oral tumors may carry an identical mutation in TP53, they are likely to have different pre-existing immune responses to their tumors. These differences may arise due to their distinct accessory mutations, genetic backgrounds, and may relate to clinical factors including previous chemotherapy exposure and concurrent medical comorbidities. In isolation, their cancer cells may respond similarly to cancer therapy, but due to their baseline variability in pre-existing immune responses, patients can have different responses to identical therapies. In this review we discuss how the immune environment of tumors develops, the critical immune cell populations in advanced cancers, and how immune interventions can manipulate the immune environment of patients with pre-malignancies or advanced cancers to improve therapeutic outcomes.

## Introduction

The local and systemic immune status of the patient plays a role in the response to conventional therapy for head and neck cancer (HNSCC). Therefore, it is critical to understand how the tumor develops both genetically and immunologically. It is in this context that this review examines the developing immune biology of HNSCC. While head and neck cancers can be found in the mucosal epithelium of the oral cavity, larynx, and pharynx, the oral cancers are predominantly associated with tobacco and alcohol use while tumors in the larynx and pharynx have been increasingly associated with HPV infection [1]. In addition, it is critical to understand whether the pre-existing immune environment can be manipulated to permit a more favorable response to treatment. From this perspective, the goal of immune manipulation is to render conventional therapies more effective in patients. Of course, it is sometimes possible to control tumors using only the immune system, but for the foreseeable future, immunotherapy will remain one element of treatment, as the fourth modality alongside some combination of the chemotherapy, surgery, and radiation therapy that is optimized to treat and cure HNSCC.

### Evidence for immune involvement in treatment outcomes

A good example of the impact of immune cells on treatment outcome is that HNSCC patients with increased immune infiltrate, particularly of T cells, have improved overall and cancer free survival. This includes oral squamous cell carcinoma (OSCC) which is typically HPV negative (HPV-) and induced by carcinogen exposure (such as alcohol and cigarette exposure) [2, 3]. In 119 patients with HPV- OSCC, increased infiltrations of CD8 T cells at the invasive margin (IM) was associated with increased overall survival following treatment [2]. In 94 patients with OSCC, a higher density of CD3^+^ T cells in both the invasive margin (IM) and center of the tumor (CT) were associated with lower stage (T1 and T2 tumors) and increased CD8^+^ and CD4^+^ T cells in the invasive margin predicted lower risk of axillary metastases [3]. In a study of 328 OSCC in the TCGA, 7 features associated with improved prognosis, both disease free and overall survival (DFS and OS, respectively), included CD8 T cells in the IM, CD45 RO in IM, CD11b in IM and CT, CD20 in the CT, FOXP3 in the CT, and PD1 in the CT. These seven features predicted better DFS and OS than other clinical and pathologic features including gender, age, smoking status, alcohol use, grade, and stage with AUC 0.755 in the discovery cohort and 0.741 in the training cohort where all other features had AUC < 0.7 [4]. These data correlate well with the earlier multiparametric analysis of immune infiltrates by immunohistology [2], where the combination of a range of immune measures including the relative location of suppressive cells generated a cumulative suppression index that showed potential to predict patient outcome [2]. In each study, the patients were treated with conventional cancer therapies, therefore the response to surgery, radiation therapy, and chemotherapy is impacted by the immune environment of the tumor at presentation.

### Opportunities to intervene to improve immune-guided prognosis

Despite this predictive and prognostic data, pre-treatment immune infiltrates do not currently alter the choice of conventional therapies given to HNSCC patients. Should we identify a patient predicted to have a poor response, we have two major options: *Option 1* is to *intensify* treatment, or, if the predictive power is sufficiently strong and negative, perhaps palliation. *Option 2* is to change the tumor so that the standard therapy becomes effective; for example, if there are few T cells in the tumor, can we not provide more? The reality is that option 2 reveals our limitations in understanding of the immune mechanisms that generate the pre-existing tumor environment at the time of treatment and in our capacity to meaningfully shape the contours of that environment for clinical benefit. There are interventions that can result in non-specific margination of T cells from the peripheral blood into peripheral tissues, such as systemic delivery of high dose IL-2. However, T cells specific for mutations in cancer are rare in the peripheral blood [5]. If the majority of T cells in the peripheral blood recognize CMV, EBV, or other common infectious agents, why would increasing the number of these cells in tumors impact patient outcome? As we will discuss, often the presence of T cell subpopulations that are enriched for tumor-specificity are more important than the infiltration of non-specific T cells into the tumor [6]. It seems rational that to meaningfully alter the outcome of a patient with poor T cell infiltration to the tumor, tumor specific T cells may need to be generated, expanded, and/or targeted to the tumor environment. Relatedly, the majority of T cells in HNSCC are found within the tumor stroma [2] rather than in contact with cancer cells, and these T cells may recirculate through the tumor environment without direct interactions with the nests of cancer cells [reviewed in Blair et al. [7]]. We may also need to consider local T cell support mechanisms such as local antigen presentation, and the differentiation signals that generate a resident tumor-specific T cell population in tumors. We should also consider the possibility that the T cells may not be the appropriate target that for manipulation to increase the accumulation of antigen-specific cells in the tumor. While this sounds counter-intuitive, tumors develop an array of immune regulatory populations that impact the ability of T cells to kill cancer cells that they recognize [reviewed in Medler et al. [8] and Tormoen et al. [9]]. Therapies that target macrophages and fibroblasts are outstanding as combination therapies in pre-clinical models, though to date they have not impacted clinical practice. These data mean that while sending a transient pulse of T cells to a tumor might increase overall infiltrates, this may not be impactful if they cannot engage with antigen, receive local cytokine support, or withstand local immune suppression that forms a critical part of the tumor stromal environment.

For example, fibroblasts that form part of the tumor stroma in HNSCC have been demonstrated to differentiate into suppressive phenotypes that associate with patient outcome [10]. While suppressive fibroblast populations were shown to impact T cell phenotypes *ex vivo* [10], cause and effect of suppressive fibroblast differentiation are difficult to separate *in vivo*, and targeting markers of specific fibroblast populations has not been impactful in our hands [11]. Fibroblast differentiation in tumors is regulated by a range of factors that also impact myeloid and T cell biology [12], and it seems likely that specific fibroblast phenotypes are reflective of broader immunoregulation of the tumor immune environment. For example, while TGFb is a potent modulator of macrophage and fibroblast phenotypes [13], genetic knockout of TGFb receptor components in different populations demonstrated that blocking TGFb responses only in T cells was sufficient to improve tumor control in pre-clinical models [14]. Similarly, cancer cells can dictate an immune environment that impacts multiple infiltrating cells *via* T cell responses. Murine HNSCC cancer cell lines selected for poor responsiveness to PD1 blockade exhibited downregulation of antigen processing and presentation, resulting in a shift from M1 to M2 macrophage differentiation and increased T reg infiltration [15]. In these models, T cell targeted interventions were sufficient to reshape the immune environment of the tumors and improve tumor control [15]. This is consistent with prior data demonstrating a reciprocal relationship between T cell and myeloid populations in tumors, and that T cell-specific immune interventions can reshape the tumor environment [16, 17]. Of course, not all immune interventions are sufficient to overcome the suppressive immune environment of tumors, and combination therapies are critical for patients that do not respond to monotherapies [18]. As we will discuss, specific immune environments of HNSCC are associated with improved outcome following conventional treatment of patients with modalities including surgery, chemotherapy, and radiation therapy [2, 3, 6, 19]. Similarly, there are immune environments that predict responses to immune therapies in many cancers, though at present few features have been validated as prognostic in HNSCC [20, 21]. We discuss how T cell targeted immune interventions can remodel the tumor immune environment such that even where they are not curative alone, they can potentially alter the response of cancer patients to conventional cancer therapies.

### Long term impact of early intervention

**Figure 1:**
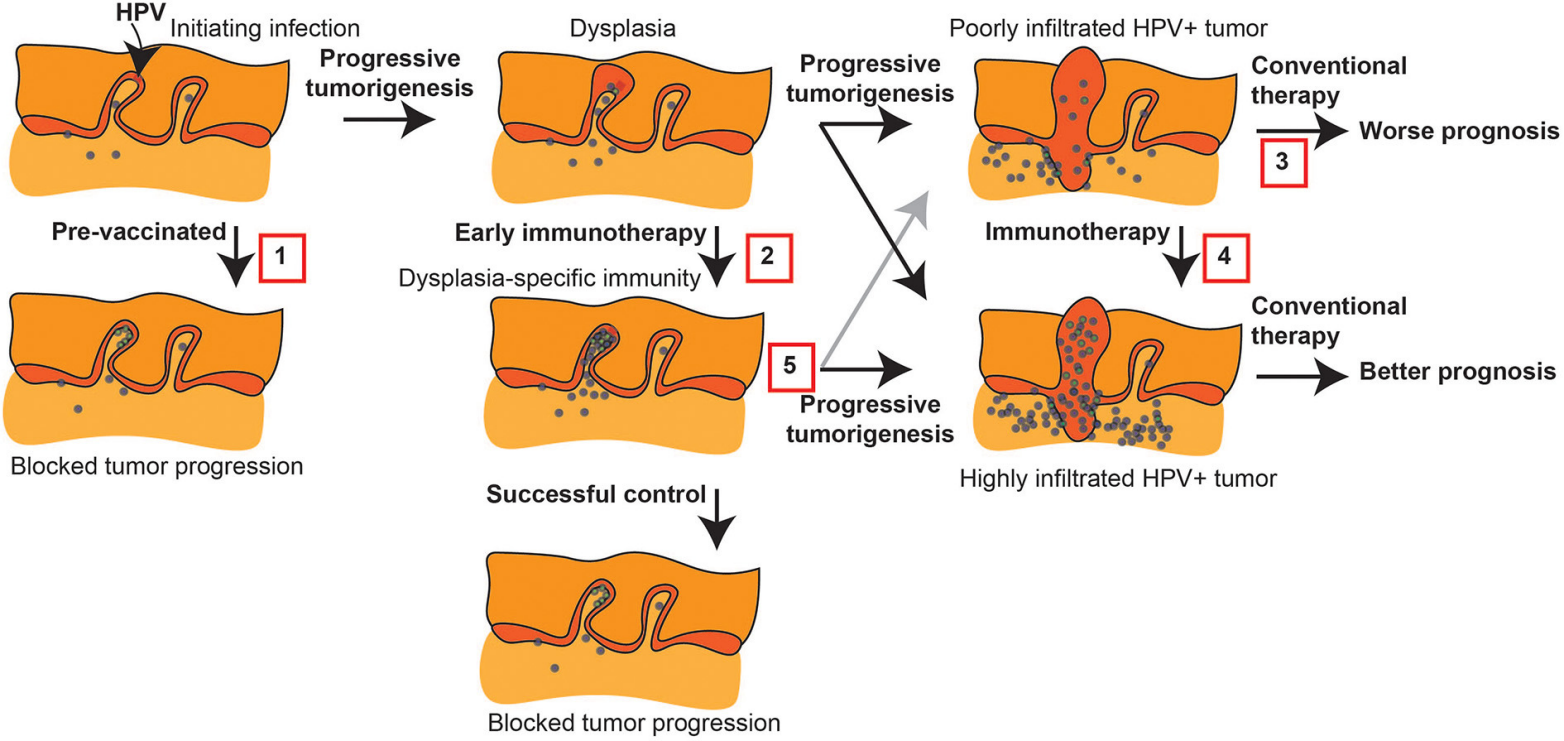
Immune intervention during tumorigenesis and prior to conventional therapy. HPV+ tumorigenesis provides a model to show potential interventions prior to conventional therapy for HNSCC that can impact outcome. 1. Protective HPV vaccination can prevent the initial transformation that can result from HPV infection. 2. Early immunotherapy of dysplasia can enhance HPV-specific responses or direct new immune responses to genes involved in progression to prevent further progression. 3. Malignant tumors can have a poor pre-existing immune response that is associated with poor prognosis following conventional therapy. 4. Immunotherapy can convert the tumor environment by directing immune responses to the tumor, and this has the potential to convert patients to an improved prognosis. 5. Even where early immunotherapy fails to prevent tumorigenesis, it may result in a tumor with a strong-pre-existing immune response that would be predicted to have a better prognosis following conventional therapy.

It is reasonable to act early to regulate the immune environment of tumors. This is most clear in HPV positive (HPV+) cancers, where preventative vaccination is successfully blocking oral HPV infection [22], and therefore has the potential to block HPV-related tumorigenesis [23]. This preventative immunotherapy can be considered as one of a range of intervention points to direct immune responses to control HNSCC (Figure 1). If preventative measures are not possible, immune therapies can be targeted to early dysplasia to prevent further transformation (Figure 1). Finally, understanding the immune features that are prognostic in patient tumors can be used to guide interventions in high-risk patients to control tumor development. Even where these interventions fail to prevent malignancies, they may result in more treatable malignancies due to their impact on the immune environment of the developing tumor (Figure 1). Many immunotherapy candidates have proven ineffective as single agent cancer therapies, but may ultimately be used to induce the changes needed to alter the immune trajectory of conventional therapy. High quality immune monitoring combined with a deeper understanding of the key features of the tumor immune environment are needed to identify such agents. We review strategies to manipulate immunity during HNSCC tumorigenesis, the critical features of the tumor T cell environment that dictates outcome in HNSCC patients, how pre-existing immune environments impact conventional cancer therapy, and how we can alter the immune environment of tumors to support novel T cell therapies.

## Manipulating immunity during tumorigenesis

### Immune environments during tumorigenic progression

As discussed above, increased immune infiltrate, particularly of T cells, are associated with improved overall and cancer free survival in patients with HNSCC [2, 3]. However, T cell infiltrates are only one feature of a complex pattern of infiltrating cells that can also impact outcome [2, 4]. The immune system's recognition of the tumor develops early in oral dysplasia and leukoplakia. In a study evaluating RNAseq for 19 patients with normal mucosa, dysplasia, and invasive tumor, immune gene expression was highest in oral dysplasia. In oral dysplasia the cytotoxic effector cell genes (including granzyme A and B) had the highest expression while in advanced disease there was highest expression of chronic inflammation (macrophages, neutrophils, and Th2 T cells) [24, 25]. There is both a developing innate and adaptive immune response starting in dysplasia that may be important in determining whether the pre-malignant lesion will progress to invasive disease. HNSCC has 4 subtypes: classical, basal, atypical, and mesenchymal that are associated with different clinical outcomes [26]. There are also two subtypes of oral dysplasia: the immune subtype is associated with an inflamed immune environment with increased CD8 T cells, monocytic dendritic cells, and macrophages and the classical subtype associated with EGFR overexpression and increased loss of heterozygosity. It is this second classical subtype, not the immune subtype, that is typically associated with progression to invasive disease and the genetic instability associated with carcinogen exposure; however, there are no specific differences in smoking or alcohol exposure between the two subtypes [27]. In progression from oral dysplasia to HNSCC, regulatory T cells, associated with immunosuppression and cancer progression, increase in proportion in the immune infiltrate [28, 29]. Oral leukoplakia is a very early oral lesion with ~3% risk of malignant transformation while proliferative leukoplakia has ~10% risk of malignant transformation. In 58 patients (29 with localized leukoplakia and 29 with proliferative leukoplakia) assessed using Nanostring digital spatial profiling, proliferative leukoplakia shows increased immune infiltrate over localized leukoplakia [30]. The proliferative leukoplakia (regardless of the degree of dysplasia) had increased expression of immune activation markers including cytotoxic T cells, B cells, and NK cells as well as increased stromal PDL1 expression [30]. In HNSCC there has been an association of FOXP3^+^ CD4^+^ T cells with improved prognosis [31, 32]. In the 4-nitroquinoline 1-oxide (4-NQO) carcinogen model of HNSCC, transiently knocking out FOXP3 cells in mice that express FOXP3 under a human diphtheria receptor caused increased T cell infiltrate in the tumor but 2.5 times increase in progression to HNSCC [33]. These data demonstrate that the immune environment of dysplasia is less immunosuppressive than invasive disease and suggest a potential pre-malignant use of immune therapies in oral dysplasia, as discussed below.

As could be expected with increased immune recognition with progressive oral lesions, increased immune checkpoint expression can be seen starting in dysplasia and increasing in invasive cancer. In leukoplakia, using the combined positive score assessment of PDL1 expression, high PDL1 expression predicted lower 5-year cancer free survival, with 70% of tumors expressing low PDL1 and 37% expressing high PDL1 [30]. While 93% (26/27) of the local leukoplakia had low PDL1, only 21% (6/28) proliferative leukoplakia tumors had low PDL1 [30]. In the 4-NQO mouse model of oral tumorigenesis, treatment with systemic anti-PD-1 immune checkpoint inhibition prevented progression [34]. Similarly, in the K14-Cre mutTP53^fl/fl^ transgenic mouse model of HNSCC treated with oral 4NQO, local delivery of anti-PD-1 immune checkpoint inhibition loaded on a hydrogel prevented progression to HNSCC [35]. Based on these data, there are several clinical trials ongoing to determine whether immune checkpoint inhibitor therapy could prevent invasive disease in patients with dysplastic lesions: NCT03692325 (nivolumab), NCT03603223 (pembrolizumab), and NCT04504552 (avelumab).

### Myeloid targets in tumorigenesis

While much of the literature has focused on the changes in adaptive immune response during progression from dysplasia to invasive disease, the innate immune response also plays a role in HNSCC development. In evaluating the immune infiltrate in the epithelium and stroma between untreated oral mucosa, dysplasia, and invasive disease in the 4NQO mouse model by RNA sequencing, the largest differences in immune infiltrate were between normal tissue and dysplasia levels of M2 macrophages (CD163+ macrophages) in both the epithelium and stroma. In normal tissue M2 macrophages were 19.1% of the immune population but in dysplasia they were 52.6%. Based on the mouse studies, an M2 macrophage gene signature was then evaluated in patients. In 86 patients with oral leukoplakia, the M2 macrophage signature was associated with improved cancer free survival [36]. The role of macrophages in progression has not been consistent in the literature because in a second study of 45 patients with oral epithelia precursor lesions and 82 patients with OSCC, the presence of M2 macrophages in precursor lesions were associated with risk of progression to OSCC [37]. The presence of another innate population, dendritic cells (DC), also is important in the tumor immune environment. DC are exclusively responsible for cross-presentation of tumor-associated antigens to CD8 T cells and can integrate innate immune signals and CD4 T cell responses to maximize tumor-specific CD8 T cell expansion. When comparing 48 oral dysplasia and 50 OSCC patients, there was a decrease in mature CD83+ Langerhans DC in the OSCC samples as compared to the oral dysplasia samples. In contrast, there was an increased plasmacytoid DC infiltrate in the OSCC as compared to dysplasia. The decreased CD83+ DC were associated with a decrease in CD8+ T cell infiltrate in the OSCC suggesting that the reduction in mature DC could result in lesser T cell recognition of the tumor and thus allowing immune escape [38]. The developing type II innate immune response and decreased mature antigen presenting cells found in the invasive cancer suggest that it is better able to escape immune detection as compared to dysplasia.

### Immune interventions to prevent tumorigenesis

With the high risk of recurrence of dysplastic lesions and risk of progression to invasive disease, vaccine strategies have been evaluated for HNSCC prevention. The vaccine strategies are separated into HPV-positive dysplasia and HPV-negative dysplasia. The HPV vaccines have been shown to prevent invasive cervical cancer in patients with cervical dysplasia and studies are currently ongoing to evaluate prevention of HNSCC in patients with HPV-positive oral dysplasia [39]. However, HPV-negative dysplasia does not benefit from this prevention therapy as there are no viral antigens to target. Tumor associated antigens including CEA, antigens identified by SEREX screens, or cancer testis antigens have been evaluated as target antigens for HPV-negative HNSCC [40]. In pre-clinical modeling, DC were pulsed with pre-malignant cell lysate and then infused in 4-NQO treated mice with oral dysplasia. The DC vaccine induced a pro-inflammatory immune response and the mice developed significantly fewer invasive lesions as compared to control mice (*p* < 0.001) [41].

In addition to direct immune targets such as PD1 as discussed above [34, 35], the cancer-targeted interventions intended to block the progression to malignancy may exploit immune mechanisms. Overexpression of EGFR can predict progression to malignancy in pre-malignant oral lesions [42]. The monoclonal anti-EGFR antibody, cetuximab, given with erlotinib for secondary prevention in patients with stage I/II HNSCC (NCT00400374) showed 71% pathologic complete response at the time of surgery, but long-term recurrence has not been reported and this combination does have considerable toxicities [43]. When erlotinib was evaluated in a randomized trial against placebo in high grade oral dysplasia (EPOC trial), there was no difference in oral cancer free survival. Despite this, there is still significant clinical and pre-clinical efforts to identify therapies for dysplasia to prevent invasive disease and improve clinical outcomes.

These data demonstrate that the immune environment of advanced cancers is developed through tumorigenesis and revealed at diagnosis. There are multiple strategies that can be incorporated to modify the contours of the immune environment during progression, which may mitigate subsequent emergence of advanced disease. Even where tumors still progress, it remains possible that those cancers that emerge may have a distinct immune profile which may alter their response to conventional cancer therapies. While it is difficult to identify tumors early, it is hopeful that the lessons from immunotherapy of pre-malignant disease may be applied to change the environment of patients with advanced cancers.

## T cell environment of responders

### Tumor-specific T cell subsets in HNSCC

As introduced above, T cell infiltration in the tumor environment is associated with better OS in oral cancers. However, initial studies did not characterize the T cell compartment to identify which T cell populations responsible for tumor growth control. Most studies have broadly focused on CD8 T cells and their role in the response against cancer. This is due in part to their capacity to recognize and kill tumor cells that present tumor antigen-derived peptides on MHC class I molecules. In oral cancers the presence of CD8 T cells at the tumor site has been correlated with better OS and improved response to check-point inhibitors. However, the CD8 T cell infiltrate is heterogenous and we have recently shown that only a fraction of the tumor-infiltrating (TIL) CD8 T cells from HNSCC patients were specific for tumor antigens [6]. Tumor-reactive CD8 T cells that co-express the surface molecules CD39 and CD103 could efficiently kill autologous tumor cells after *in vitro* expansion [6, 44]. CD8 T cells with a similar phenotype were also observed in other tumor histologies such as melanoma, breast cancer, colorectal cancer, and non-small cell lung cancer [6, 45]. Expression of CD103 and CD69 and lack of CCR7 expression by CD39^+^CD103^+^ CD8 TIL favors their tumor residency [6, 44] and this is highlighted by little overlap between the TCR repertoire of CD39^+^CD103^+^CD8 TIL and circulating memory CD8 T cells [6]. In addition, an increased frequency of CD39^+^CD103^+^ CD8 cells among total tumor infiltrating CD8 T cells at time of surgery correlated with a better OS in a cohort of HNSCC patients [6]. Together, these results imply that CD39^+^CD103^+^CD8 T cells in the tumor are involved in the anti-tumor immune response. Therefore, gaining insight about the development requirements, maintenance, and cellular interactions of these cells in the tumor might provide new avenues for the treatments for cancer. In addition, understanding the changes in this CD8 T cell compartment between dysplasia and invasive cancer would provide valuable information for future interventions.

A recent study in HPV-positive HNSCC patients helps demonstrate the regulation of these cells [46]. Using tetramers to isolate HPV-reactive CD8 T cells, which all expressed PD-1, the investigators found that HPV-specific T cells were composed of three transcriptionally distinct cell clusters: a stem-like cluster; a transitory cluster; and a terminally differentiated cluster [46]. While the stem-like subset was characterized by the expression of *TCF7* and *IL7R*, the transitory subset expressed *PRDM1* and *IFNG*, and the terminally differentiated subset expressed *HAVCR2, GZMA, GZMB*, and *PRF1*. Following cell sorting and *in-vitro* peptide stimulation, cells from the stem-like subset but not the terminally differentiated subset were able to proliferate and differentiate into effector-like CD8 T cells [46]. Cells from the other subset maintained their terminally differentiated phenotype. Thus, an efficient anti-tumor immune response might require controlling the balance between those three subsets to maintain a pool of effector CD8 T cells capable of recognizing and lysing tumor cells. It will be important to determine whether a similar pathway exists for non-viral tumor antigens such as neoantigen-reactive CD8 T cells and understand if this mechanism is influenced by the affinity of a TCR for its cognate epitope.

### Role of CD4 T cell help

Even though HPV-driven oral cancers have a better prognosis than HPV-negative tumors, in our experience there is no significant difference in the frequency of CD39^+^CD103^+^CD8 T cells among total CD8 T cells infiltrating the tumor between those two groups of patients (unpublished data). This observation suggests that other components of the immune environment influence the biology of tumor-reactive CD8 T cells. Indeed, it is known that CD8 T cells need to receive signals from CD4 helper T (Th) cells to become fully licensed to kill target cells [47]. Strong infiltration by CD4 T cells in oropharyngeal squamous cell carcinoma (OPSCC) was associated with lower T stage, improved disease specific survival and prolonged overall survival supporting a role for these cells in the anti-tumor immune response [48, 49]. However, these data did not address the complexity of the CD4 compartment and the role of the distinct CD4 T cell subsets in this effect. Similar to their frequency in the peripheral blood, CD4 T cells account for a large portion of the T cells in the tumor. Among the CD4 T cells infiltrating HNSCC, 30–40% of the cells are Treg cells [50], involved in regulating immune responses, preventing immune pathologies, and thus limit anti-tumor immune responses. In breast cancer and NSCLC, CCR8 expression identifies a subset of highly immunosuppressive Treg cells which also express CD39 and ICOS [51, 52]. The remainder of the intratumoral CD4 T cells include distinct cell subsets specialized for immune responses against different immunological insults and orchestrating both cellular and humoral immune responses to infectious agents and cancer [53]. Each subset is characterized by a master transcription factor and the secretion of hallmark cytokines. However, there is only scant data identifying the Th composition in oral cancers. Most of the TIL CD4 Th cells have an effector/memory phenotype. Among those, some express high levels of the activation/exhaustion markers PD-1, HLA-DR, CD39, and CTLA-4 [50, 54, 55]. In contrast to the CD8 compartment, CD103+ CD4 Th cells are rare [19, 55], suggesting distinct requirements between CD4 and CD8 T cells for CD103 expression.

The comparison of HPV+ and HPV-HNSCC tumors by single cell RNAseq analysis has shown differences in the CD4 compartment [54]. HPV+ tumors showed an enrichment in cells with a follicular CD4 Th (Tfh) cells or Th1 gene signatures, whereas HPV-negative tumors were composed mostly of effector or effector/memory CD4 Th cells [54]. The observed difference regarding Tfh cell signature reflects the higher frequency of B cells in HPV+ HNSCC as compared to HPV- HNSCC [54, 56, 57]. Alternatively, the data could be explained by the different anatomical locations between those two types of HNSCC, where HPV+ HNSCC exists as part of the oral ring of secondary lymphoid organs. In a mouse model of NSCLC, the presence of both neoantigen-reactive CD4 Th cells and B cells in the tumor was shown to be beneficial for the anti-tumor immune response [58]. Furthermore, interactions between those two cell types were necessary for the generation of IL-21-producing Tfh cells that promoted anti-tumor immunity by enhancing CD8 T cell effector functions [58]. Tfh-like CD4 T cells characterized by the secretion of the B cell-attracting CXCL13 have also been identified in breast cancer and their frequency correlates with better outcome [58]. More recently, we reported the presence of a subset of CD4 Th cells that co-express PD-1 and ICOS, a phenotype reminiscent of Tfh cells, in the tumor microenvironment (TME) of HNSCC tumors [19]. Those cells also expressed the transcription factor BCL6 and secreted IL-21 and CXCL13. More importantly, PD-1+ICOS+ CD4 Th cells were enriched in T cells recognizing tumor-associated antigens such as HPV but also tumor specific neoantigens. Future studies are needed to confirm whether PD-1+ICOS+ CD4 Th cells interact with B cells and CD8 T cells in the TME. Such experiments are important to distinguish the true function of CD4 Tfh and B cells in HNSCC, since they may be a secondary feature of tumor immune biology. Tertiary lymphoid follicles have been detected in a wide array of cancers [59], including poorly-immune responsive tumors [60]. Their formation has been linked to inflammatory conditions in multiple disease and may be a feature of inflammatory tumors rather than a cause of anti-tumor immunity. It is unclear whether T cells in the tertiary lymphoid structures can emigrate into the tumor, or must follow lymphoid drainage and rely on recirculation to re-enter the tumor environment. Spatial analysis of the TME will be necessary to confirm the cellular interactions of CD4 Tfh and CD8 in human tumor specimens. As discussed earlier, multiparametric analysis of immune infiltrates by immunohistology [2], demonstrated that the relative location of CD8 T cells in relation to CD4 Treg formed part of a cumulative suppression index that showed potential to predict HNSCC patient outcome [2]. Interestingly, in patients with pancreatic cancer, tumors with tertiary lymphoid structures were less enriched for tumor-infiltrating CD103^+^ cells [60], suggesting that the biology driving expansion of CD4 Tfh in tumor follicular structures may be different from that driving Trm. Within the tumor, T cells are generally enriched in the tumor stroma, and less frequent in cancer cell nests [61–63]. Cells with the Trm phenotype are enriched in cancer cell nests rather than the tumor stroma in breast cancer patients [63], while stem-like CD8 T cells expressing the Tcf1 marker were located in the stroma and absent from cancer cell nests in melanoma patients [64]. Further studies are needed in HNSCC to confirm these data, but these data suggest that Trm exist near cancer cells, while tumor specific T cells with other phenotypes recirculate through the tumor stroma [7]. Along with spatial information, it will also be important to understand the molecular signals required to induce the recruitment and differentiation of CD4 Th cells into Tfh-like CD4 T cells in the tumor environment. Finally, it will be essential to determine if the Tfh-like CD4 T cells can directly recognize tumor antigens in HNSCC patients, since this will directly relate to the specificity of B cells that they support and the CD8 T cells that they might help. These data indicate that specific T cell subtypes in HNSCC impact patient survival. Given our new tools to identify and characterize tumor-specific T cells through a combination of epitope prediction, functional analysis, and single cell RNASeq, we can anticipate rapid advances in our understanding of the critical tumor antigen-reactive T cells in HNSCC.

## Impact of immunotherapy on pre-existing or new responses to tumor antigens

### *In vivo* initiation of immunity

**Figure 2:**
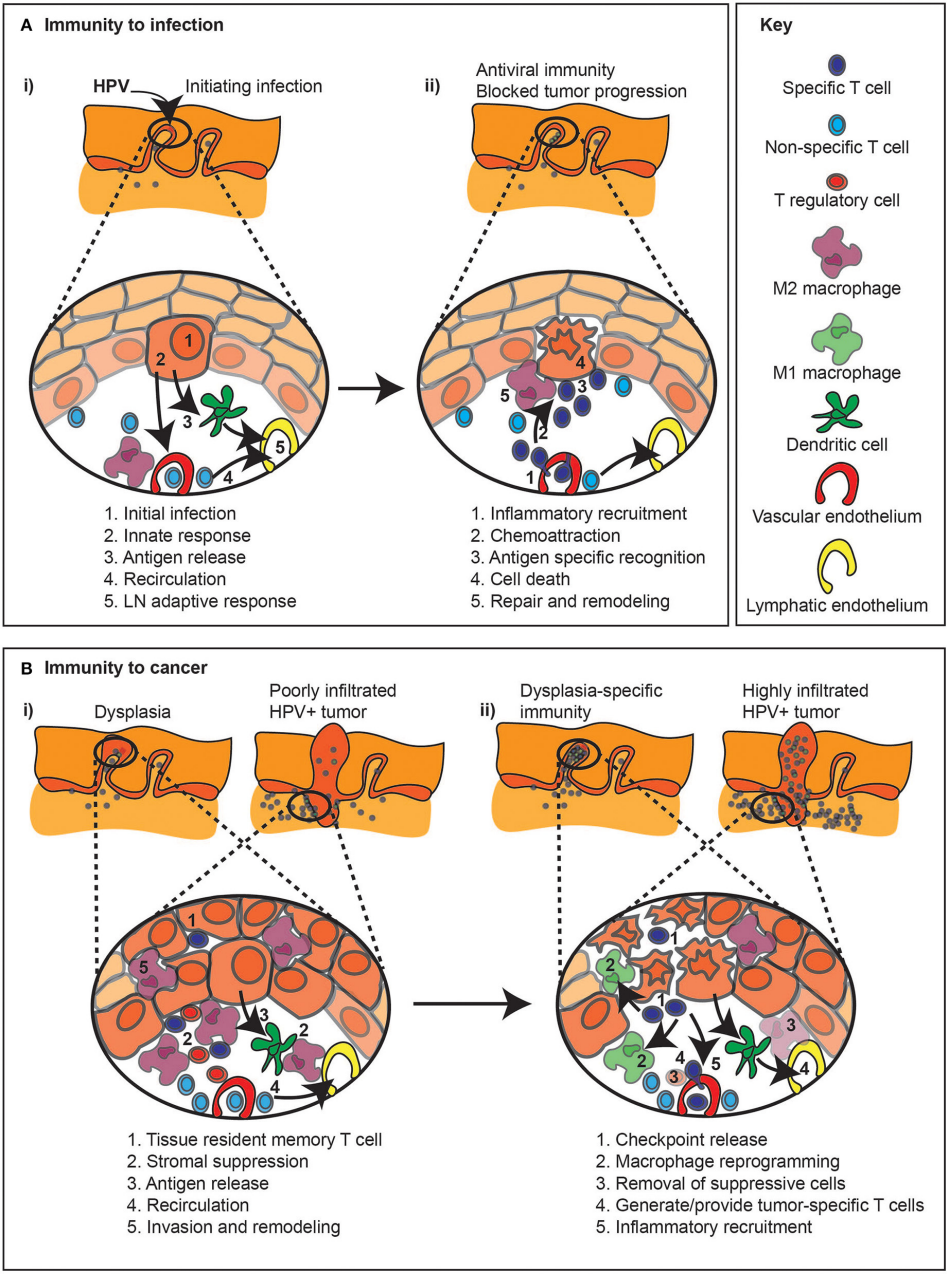
Immune regulation during infection and related immunotherapy effects on pre-existing anti-tumor immune responses. **(A)** (i) The response to an infection, in this example HPV infection of basal epithelial cells (1), can result in a series of events that result in immunity. Innate sensing of infection (2) can lead to locoregional inflammation (2), as well as antiviral responses in the infected cell. Inflammation combined with antigen release can permit DC migration to lymph nodes (3), so while non-specific cells continue to recirculate through the tissue (4), new immune responses can be initiated in the draining lymph node (5). (ii) Following expansion of antiviral T cells in the draining lymph node, these cells can be preferentially recruited to the site of infection *via* the impact of inflammation on the vasculature (1). Chemokines released by infected cells can recruit T cells through the stroma to the infection site (2). Cognate recognition of infected cells (3) can lead to their death (4). Dying cells are actively cleared, which can initiate repair and shut down inflammation in the infection site (5). **(B)** (i) The immune response to cancer, either in early dysplasia or in a malignant tumor, can result in infiltration of tissue resident memory T cells (1), as well as stromal infiltration of T cells, though local immune suppression (2) results in tumor growth dominating over tumor destruction. Ongoing cancer cell death, due to hypoxia or limited growth factor availability can lead to antigen release (3), though stromal suppression can limit the quality of antigen presentation. T cells can continue to recirculate through tumors (4), though they may be restricted to the stroma and poorly interact with cancer cells, and stromal cells can support further invasion of the cancer into surrounding tissues (5). (ii) Immunotherapy with checkpoint inhibitors (1) may derepress local T cells or recirculating T cells to permit destruction of cancer cells. Inflammatory cytokines released by activated T cells can repolarize macrophages in the tumor to limit invasion or immune suppression (2). Alternatively, immunotherapies can be selected to deplete or block the inhibitory effect of suppressive macrophages or Treg (3). Where pre-existing immunity is limited, vaccine or adoptive transfer approaches may provide tumor-specific T cells (4), which can be targeted to the tumor environment with locoregional immunotherapies that generate inflammation in the tumor environment (5).

Classical responses to exogenous infection, for example immunity to an infecting virus, depends on an efficient transition from innate to adaptive immunity. Initial infection of HPV into basal cells in the tonsillar epithelia, can result in innate immune detection of the invading virus (Figure 2A). These innate signals can result in local immune activation, such as STING-mediated nucleic acid sensing resulting in type I IFN production [65]. Innate immune responses can result in local dendritic cell maturation and trafficking to draining lymph nodes to initiate anti-viral responses and activate the vascular endothelia to encourage infiltration of T cells to the infection site (Figure 2A). Successful antiviral immunity can result in destruction of infected cells. Unfortunately, HPV has a series of evolutionary adaptations to block various aspects of innate and adaptive detection [65–67] permitting immune escape. These immune evasion mechanisms can allow tumor initiation and limit immune responses in tumors (Figure 2B). While there can be tumor-specific T cells in pre-malignancies and malignant tumors, there are many ways that the tumor environment can suppress adequate anti-tumor immune function, including expression of checkpoint regulators, suppressive macrophages and Treg (Figure 2B) and these features can support tumor growth despite the existence of tumor-specific T cells. As we will discuss, interventions that form the basis for current immunotherapies focus on releasing existing cells from checkpoint inhibition, deplete or reprogram suppressive cells, and generate local inflammation to increase immune function in the tumor (Figure 2B). However, in patients with poor pre-existing immunity to their tumor, alternative immune interventions such as adoptive transfer or vaccination may be necessary to provide new cells to target the cancer cell. Removal of a negative feature, such as immune suppression, can only have impact if there are pre-existing anti-tumor immune responses waiting to become active.

### Evidence for pre-existing immunity determining therapeutic responses

In pre-clinical models, the development of tumor-specific T cells following tumor implantation is dependent on a fully functional immune system [reviewed in Medler et al. [8]]. A failure in any part of that system can result in abnormal immunity. For example, mice deficient in cross-presenting DC *via* a Batf3 knockout fail to develop functional T cell infiltration in tumors [68]. DC are key for tumors to develop an immune infiltrate even before the tumors become detectable in mice. The degree to which tumors engender T cell immunity early in their development can directly impact their ability to grow in immune competent mice [8, 68]. We discussed this impact for progressing pre-malignant tumor models above. For transplantable tumor models, the injection of cancer cells into mice acts as an initial tumor-specific vaccination event resulting in CD8 T cell immunity which is closely followed by T regulatory cell suppression [69–73]. Thus, even in models that do not progress through pre-malignancy, early immune activation dictates the eventual immune environment of the tumor. This directly impacts treatment responses. For example, we previously demonstrated that the response to combination radiation therapy and checkpoint inhibitor was dependent on the presence of a pre-existing immune responses developed at tumor implantation [74]. When implantation immunity was blocked, these therapies were no longer effective. Therefore, while the goal of combination immunotherapy with radiation therapy has been assumed to result in antigen and endogenous adjuvant release resulting in *de novo* immune responses to tumors, in our studies the treatment expands existing responses essential for improved outcomes [74]. When considering checkpoint inhibitor immunotherapy this is logical, since checkpoints such as PD-1 and CTLA4 are expressed on antigen experienced T cells. It is reasonable that these therapies remove suppression of an existing population of currently suppressed cells, therefore are dependent on pre-existing immune responses. This is consistent with data in pre-clinical models, where tumor control by combination checkpoint immunotherapy was shown to be dependent on T cells already residing with the tumor, and is lost if immune responses at tumor implantation are blocked [75]. Checkpoint inhibition may not act on all suppressed cells in the tumor, since some may not easily be restored to full function. The phenomenon of tumor-specific T cell exhaustion, akin to that observed in chronic antiviral responses [76], renders T cells unresponsive to formerly effective stimuli [77]. T cell exhaustion has been demonstrated early in tumor progression [78], with exhaustion irreversible by the time solid tumors are detected [78]. As discussed above these T cell populations are very relevant to HNSCC [46] and recent data suggests that terminally exhausted cells in the tumor cannot be restored to full function by checkpoint inhibition. Instead, therapy allows a pre-existing progenitor exhausted population to participate in tumor control [64, 79]. These data suggest that immunotherapy recruits additional immune cells, present in the tumor or recirculating but currently unable to impact tumor growth, to participate in immune control of tumors.

### Therapeutic interventions to bring new responses to the tumor

**Figure 3:**
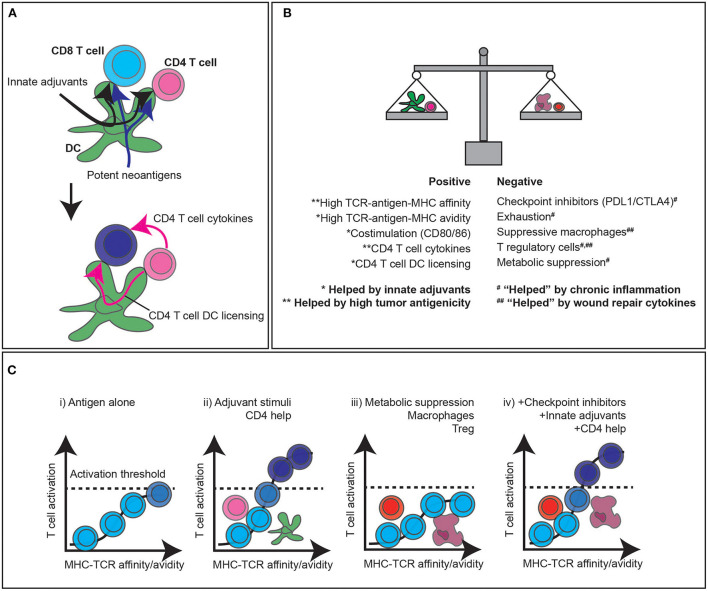
CD4 T cell help, adjuvant signals, and immunotherapy to enhance CD8 T cell responses to tumors. **(A)** The classic three cell model of CD8 T cell activation relies on antigens being presented to both CD4 and CD8 T cells by dendritic cells. Innate adjuvants present in the infection improve antigen processing and presentation, as well as upregulate costimulatory molecules. Highly antigenic targets increase the likelihood of high affinity antigens being available for both CD8 targeting and CD4 T cell help. **(B)** The balance of positive and negative stimuli dictate whether T cells can contribute to anti-tumor responses. Broadly, highly antigenic tumors with endogenous adjuvant support can improve the likelihood of T cell responses, which are countered by a range of negative regulatory features of the tumor environment. **(C)** High affinity antigen-specific T cell responses have the potential to stimulate T cells independently, but the likelihood of passing the critical activation thresholds is increased by CD4 help and adjuvant signals to cross-presenting DC and directly presenting cancer cells. A range of suppressive factors in the tumor can decrease the ability of T cells to pass the critical activation threshold to control tumors, and current immunotherapies can overcome these limitations to overcome suppression and bring new T cells into the response that were formerly unable to cross the activation threshold to participate in tumor control.

Of course, antagonist anti-CTLA4 and agonists such as anti-OX40 can act as vaccine adjuvants expanding *de novo* T cell responses following antigen challenge [80–82]. Thus, aside from their effect on existing T cells, there is the potential to bring new cells into the immune response at the tumor. Given the long exposure of patients to tumor-associated antigen, it is unclear whether there exist naïve T cells specific for tumor-associated antigens that have managed to avoid meeting this antigen in their recirculation through lymph nodes. However, it is reasonable that T cells with TCR affinities that are too low for them to functionally participate in endogenous anti-tumor immunity are a potential new source of anti-tumor immunity. For these cells to participate, they need the threshold for activation to be manipulated in their favor (Figure 3). To trigger full activation of CD8 T cells, both the amount of presented antigen and the affinity of the TCR for the antigen-MHC combination matter [83, 84]. This interaction is helped by innate adjuvants, which can increase antigen processing and presentation and upregulate costimulatory molecules such as CD80 and CD86 [85–89] (Figure 3). In addition, successful antigen presentation to CD4 T cells can help CD8 T cell responses, both through cytokines and DC licensing [90–92]. The limiting effect of low TCR affinity for tumor-associated antigen has been demonstrated [93] and results in T cell dysfunction. Importantly, both anti-PD1 and anti-CTLA4 overcome suppressed TCR signaling through effects on phosphatases such as Shp2 and PP2A [94–97] and availability of costimulatory signals [98, 99] altering the signaling threshold of T cells. In pre-clinical models, tumor control with antigens that were suboptimal at engaging T cells is improved on treatment with anti-PD1 and anti-CTLA4 [93]. TGFb acts in part to alter the signaling threshold of T cells, and TGFb inhibition allows T cells to fully activate with normally suboptimal targets [14, 100]. Similarly, novel regulators such as PTPN22 are emerging that can be targeted to improve TCR activation and enhance tumor immune responses [101]. These data mean that even without generating truly *de novo* immune responses to tumor-associated antigens, our most popular cancer immunotherapies serve to bring suboptimal T cells into the anti-tumor immune response. These data indicate the importance of regulating the activation of pre-existing T cells and improving the response of sub-optimal T cells that were formerly unable to functionally participate. In addition to exploiting pre-existing immunity to alter immune responses to growing tumors, we can restore the function of failed responses to expand tumor immune control. As we will discuss, this may in part account for the beneficial effect of autologous/expanded TIL therapy, and/or repeated infusion of expanded TIL-derived cell products [102].

## Adoptive T cell therapies to overcome suboptimal pre-existing immunity

### Adoptive TIL-based T cell therapy platforms in HNSCC

The initial success of autologous TIL therapy in melanoma relied on access to rIL-2 in quantities necessary for clinical use [103–105] and was then enhanced further by incorporation of preparative non-myeloablative (NMA) conditioning [106–109]. The mechanisms of this therapy appear to rely on optimizing transferred T cell expansion *in vivo* to generate diverse T cell phenotypes that can overcome ongoing T cell dysfunction in the patient [110, 111]. This established a standard three-step therapeutic sequence for conventional TIL therapy, consisting of: (i) conditioning; (ii) cell infusion; and (iii) cytokine support. Despite these successes in melanoma, successful experiences with TIL therapy have been limited in epithelial malignancies, including HNSCC. For example, in 2015 through an institutional research protocol, we identified a 21-year-old patient with treatment-refractory recurrent OSCC, for whom *ex vivo* cultured TIL showed a high degree of reactivity and specificity by *in vitro* assay to his autologous primary tumor cell line, but not to HLA-matched, allogeneic tumor cell line controls. Under an FDA single-patient IND, autologous TIL were expanded under cGMP conditions and he was treated with standard cyclophosphamide/fludarabine NMA conditioning followed 1 week later by cell infusion and high dose IL-2 support, but unfortunately, he did not respond to therapy [112]. In late 2018, Iovance released preliminary results from a phase II trial of conventional TIL therapy, including cyclophosphamide/fludarabine NMA conditioning and high dose IL-2 support (LN-145) in 2nd line or later relapsed and metastatic HNSCC (NCT03083873) [113]. Patients had a median of 3 prior therapies. Responses were observed in 4 of 17 patients (anatomic subsite and HPV-status not reported). A follow-up Iovance phase II trial of LN-145 plus a concurrent single dose of pembrolizumab, in immunotherapy-naïve relapsed and metastatic (r/m) HNSCC, (NCT03645928), reported initial results in late 2021 with confirmed partial or complete (PR and CR, respectively), in 5 of 18 patients, and unconfirmed clinical response in an additional two patients, for overall response rate of 39% [114]. Patients had a median of one prior line of therapy. Again, anatomic subsites and HPV-status were not reported, so it is unknown if responses included OSCC. It is plausible that responses were primarily in HPV-associated cases [115–118].

### TCR-Transduced adoptive T cell therapy in HNSCC

Promising results from an NCI group, first reported in late 2018, used a gene-modified TCR-transduced adoptive T cell therapy (TCR-T) targeting HPV16 E7 protein [119–122] viral tumor-associated antigen (HLA-A^*^02:01 or A^*^02:06 restricted) for T cell mediated eradication of HPV16-associated tumors (NCT02858310) [123]. Complete regression of most tumors was observed with 6 of 12 treated patients. The majority of these patients were refractory to prior PD-1 blockade including all the HNSCC patients (ORR 50%, all PR/no CR) [124]. Among the four HNSCC patients, two responses were observed, including one patient who had been previously treated on the Iovance trial of LN-145 conventional TIL therapy at our center. Intra-patient tissue genomic analysis representing dichotomous tumor responses, revealed known resistance mechanisms involving defects in antigen presentation and interferon response pathways consistent with immune editing.

In 2020, Adaptimmune reported the ADP-A2M4 single-arm, phase II pilot trial of MAGE-A4 targeted, HLA-A^*^02:01 restricted TCR-T therapy in combination with pembrolizumab for first-line recurrent/metastatic, HPV-agnostic HNSCC. This trial has since closed due to slow accrual of MAGE-A4 expressing & HLA-matched eligible participants (NCT03132922) [125]. In 2021, Immatics reported preliminary results from the IMA203 phase I dose-escalation trial of MAGE-A4/A8 (PRAME) targeted, HLA-A^*^02:01 restricted TCR-T therapy for treatment-refractory advanced solid tumors (NCT03686124) [126] at the SITC annual meeting. Of 16 participants, three HNSCC patients were treated, but none with durable response more than 12 weeks (subsite and HPV-status not specified, but OSCC eligible). At this time, both companies are moving forward with next generation TCR-T products incorporating a CD8a coreceptor and affinity optimized TCR [127, 128], OSCC patients may be eligible in future trials given prior interest in HNSCC for MAGE-A4 targeting.

### Chimeric antigen receptor T cell therapy in HNSCC

Systemic delivery of CAR-T targeting HNSCC surface antigens, to date been shown to be unsafe due to the unacceptable risk of on-target/off-tumor toxicities [129, 130]. CAR-T may be inferior to TCR-T in the long-term, due to more rapid exhaustion, despite more potent initial effector and killing function [131–133]. However, CAR-T intratumoral delivery in OSCC may hold promise as a means of TME remodeling and immune priming. A phase 1 dose-escalation, single center trial of intratumorally delivered pan-ErbB CAR-T in HNSCC (NCT01818323) [134] was reported at AACR in 2017 [135] and at ASCO in 2018 [136], using T1E28ζ, a CAR containing a promiscuous ErbB ligand (which engages 8/9 ErbB homo/heterodimers) coupled to a CD28 + CD3ζ endodomain and 4αβ, an IL-4-responsive chimeric cytokine receptor which enables IL-4-driven selective CAR T-cell enrichment/expansion during manufacture [137]. CAR T-cell dose was escalated from 1 × 10e7 to 1 × 10e9 T-cells administered as a single treatment, by multifocal intra-tumoral injection without lymphodepletion and no dose limiting toxicities were observed. Three observations in this study point to tumor remodeling effects: (i) tumor growth slowed to stable disease in 10 of 16 patients, despite rapid disease progression on study entry; (ii) CAR-T cells remained undetectable in the circulation throughout; and (iii) a rapid complete response was observed in a patient who subsequently received aPD-1 + T-VEC, since durable for >3 years.

### Additional modification of T cell therapy platforms

**Figure 4:**
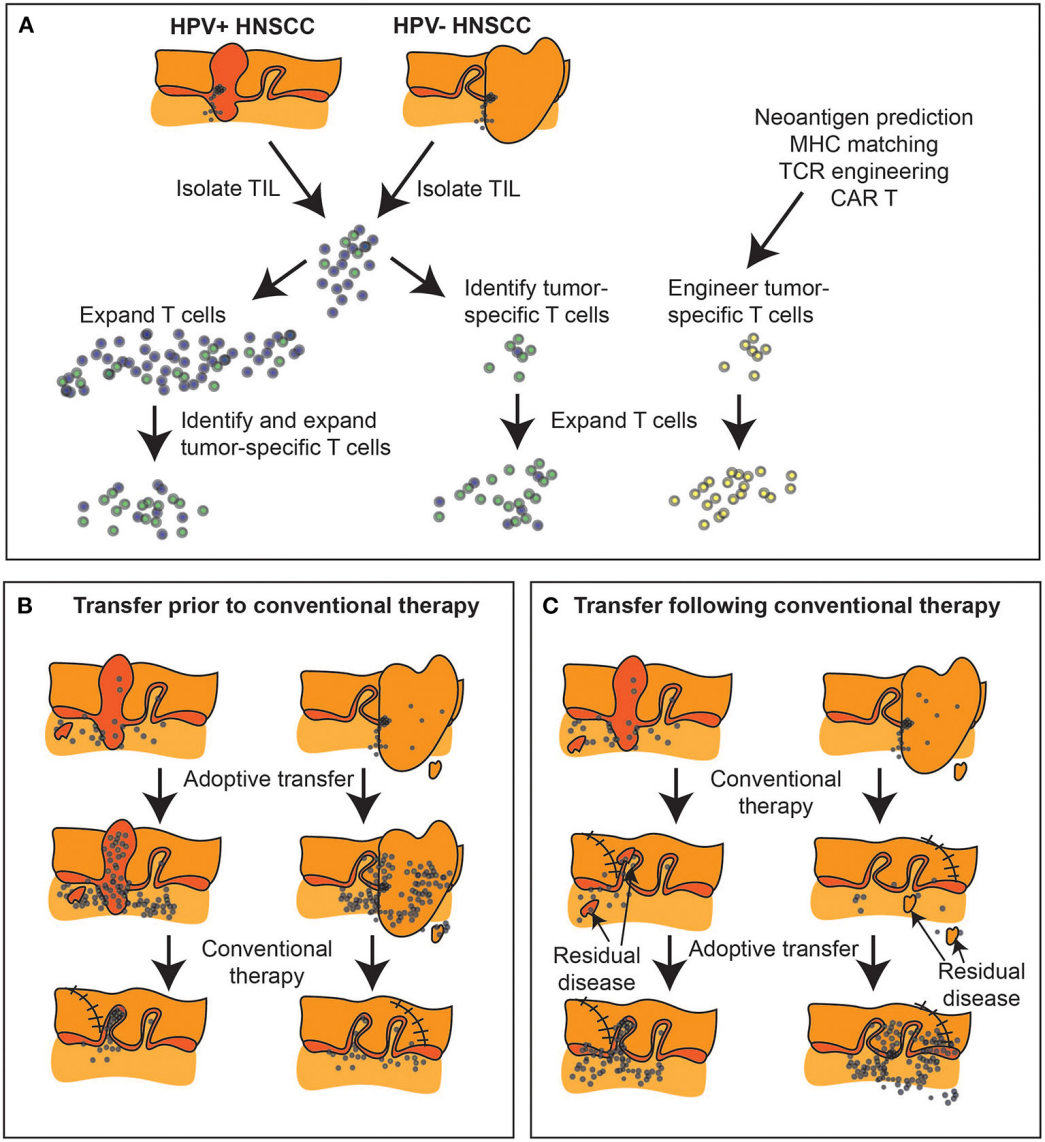
Expanding tumor-specific T cell immunity *via* adoptive transfer. **(A)** Biopsies and surgical specimen can be a source of tumor infiltrating lymphocytes (TIL), which can be expanded *ex vivo* and tumor-specific cells isolated either by response, or by initial phenotype. Alternatively, tumor-specific T cells can be engineered if both antigen and MHC match known TCR specificities, or using chimeric antigen receptors (CAR). **(B)** Adoptive transfer can occur prior to conventional therapy to alter the tumor immune environment prior to further treatment, or **(C)** can follow conventional therapy to target residual disease. At present, with surgical samples the dominant source of TIL, adoptive transfer is generally delivered following completion of conventional multimodality treatment or in the metastatic setting.

With the advent of widely available tumor sequencing, tumor neoantigen redirected T cell therapies [138] (such as TCR-T) hold enormous promise for truly tumor-unique targeting without off-tumor/on-target concerns (Figure 4A). These have already demonstrated initial, though limited, success in multiple epithelial malignancies [139–143] including cholangiocarcinoma [102], colon [144, 145], and breast cancer [146, 147]. Similarly, we recently demonstrated the potential of KRAS-specific TCR-T for pancreatic cancer [148]. By extension, HRAS mutations have been identified as a conserved neoantigen potential target for redirected T cell therapy in OSCC [149]. Numerous synthetic biology approaches are being brought to bear in order to enhance cell-intrinsic characteristics and cell product manufacturing [142, 150] including: selection of optimal T cell subpopulations for transduction (including non-viral transduction); manipulation of cell culture to promote favorable phenotype and improve yield during the manufacturing process; transduction of druggable growth receptors to introduce control of *ex vivo* and *in vivo* proliferative kinetics; TCR affinity and signaling enhancements, membrane-anchored cytokines to promote *in vivo* persistence; conversion switches to couple an inhibitory cell surface receptor with a stimulatory cytosolic tail, and thus armor the cells for trafficking, survival and effector function on entry to a hostile TME. These cell-intrinsic enhancements undoubtedly will increase therapeutic efficacy. Nevertheless, the foremost challenge to TCR-based cell therapy approaches remains ineffective antigen presentation without which T cell-dependent immunotherapy, whether through immune checkpoint inhibitor or adoptive cell therapy, cannot exert an effect. MHC class I down-regulation and alterations in antigen processing and presentation are prevalent in HNSCC (selective HLA loss of 37% in primary lesions; total HLA loss of 15% in primary lesions and 40% in metastatic lesions) [151] and are well-described [152–158]. Several groups have demonstrated that MHC class I down-regulation portends a poor prognosis in OSCC specifically [2, 159] and in HNSCC more broadly [160–162] and represents a mechanism for immune escape and acquired resistance to immunotherapy, generally [163]. NK cell-based therapies, which can detect and eliminate tumor cells *via* MHC-independent mechanisms, have been proposed to overcome this challenge in HNSCC. NK-92 cells [164] engineered to express endoplasmic reticulum-retained IL-2 [165] and a second generation CAR targeting PD-L1 [166] (PD-L1 t-haNK) were shown by the NIDCD group to eradicate MOC1 tumors *in vitro* in a PD-L1-dependent fashion [167]. In an OSCC model system of adoptive T cell therapy immune escape, using UM-SCC-1 and UM-SCC-47 oral cancer cell lines with varying admixtures of HLA-expressing and HLA-nul tumor cells, the addition of PD-L1 t-haNK *in vitro* was able to salvage response and prevent clonal outgrowth of escape variant tumor cells [167]. The NIDCD group further demonstrated synergy of combinatorial PD-L1 t-haNK, PD-1 blockade, and IL-15 superagonist in the MOC1 syngeneic mouse oral cancer model laying the groundwork for a recently activated phase II trial (*n* = 55) of this triplet combination in 2nd line recurrent/metastatic HNSCC (NCT04847466). A potential advantage of CAR-NK cell therapy over CAR-T therapy is a very reduced incidence of graft vs. host disease due to limited longevity. Tracking studies indicate that PD-L1 t-haNK cells persist in tumors for up to 72 h, suggesting that to ensure the maintenance of cells *in situ*, weekly or more frequent dosing may be necessary.

### Targeting the tumor immune environment to enhance adoptive transfer

Myeloid cells represent a potential impediment to effective adoptive cell therapy approaches in OSCC [168]. In HNSCC patients an expanded population of CD34+ myeloid cells that can suppress T cells has long been identified [169, 170], which match the consensus definition of myeloid-derived suppressor cells [171]. Using murine oral cancer (MOC) C57BL/6 syngeneic tumor models, PI3Kδ/γ inhibition or anti-Ly6G depletion inhibits the impact of the expanded myeloid populations and enhances responses to PD-L1 mAb in T-cell–inflamed MOC1, but not in non-T-cell–inflamed MOC2 tumors [172]. Similarly, using the same models anti-Ly6G depletion was shown to improve tumor control following anti-CTLA-4 treatment in T-cell inflamed MOC1 tumors, but again not in non-T-cell inflamed MOC2 tumors [173]. Recent studies demonstrate that SX-682, an oral CXCR1/2 inhibitor, disrupts MDSC trafficking, and improves tumor control by anti-PD1 therapy in an oral cancer model [174], as previously shown in other tumor models [175, 176]. These data demonstrate that where T cells are abundant but suppressed, myeloid targeting may optimize T cell control of tumors, though in poorly infiltrated tumors there may be limited benefit. However, this blockade of CXCR1/2 also enhances tumor infiltration, activation, and therapeutic efficacy of adoptively transferred murine NK cells [177], suggesting that cellular therapies can be improved by therapeutic modulation of the tumor environment. In addition, cellular therapies may be able to directly deplete these expanded myeloid cells, since the PD-L1 targeting CAR-NK (PD-L1 t-haNK) was able to preferentially lyse MDSC in the PBMC fraction of peripheral blood cells [166]. Thus, in addition to targeting PD-L1-expressing cancer cells [166], the on-target off-tumor lysis of normal cells may modify the tumor immune environment.

### Immunotherapy combinations for adoptive transfer

Under the conventional three-step therapeutic sequence of TIL therapy (conditioning/cell infusion/cytokine support), it is worthy to note here that the non-myeloablative conditioning regimen of cyclophosphamide/fludarabine (Cy/Flu)—which has modest activity in HNSCC [178, 179]—has been unchanged for decades. This represents an area of opportunity for remodeling of the tumor immune microenvironment in the current context of multiple bioactive immune modulating agents that are newly available or in clinical development. This can be put to the test, either alone or in combination with chemotherapy, as immune induction during the existing preparative window preceding cell infusion. The observation that CD40 signaling in APCs may remodel the TME [180] and that effective T cell therapy results in T cell-directed and CD40-mediated remodeling of the tumor stroma [181], suggests that this approach has potential to alter the contours of the tumor immune environment. In pre-clinical models, CD40-CD40L interactions were necessary to develop pre-existing T cell responses in tumors and therefore essential for the response to immunotherapy and conventional radiation therapy combinations [74]. In pre-clinical models CD40 agonist antibodies can enhance the expansion of adoptively transferred T cells *in vivo* and boost their antitumor activity [182]. Using the KPC model of intraepithelial pancreatic tumorigenesis, cDC1 dysfunction and apoptosis was shown to be neutralized *in vivo* by IL-6 blockade, and CD40-agonist restored cDC1 maturation and abundance, resulting in enhanced control of tumor outgrowth [183]. Standard conditioning Cy/Flu lymphodepletion was observed to induce peripheral MDSC expansion in melanoma patients treated with TIL therapy [184], and was associated with reduced TIL persistence and poorer survival. In the B16/pmel-1 model, IL-6 driven differentiation of mobilized hematopoietic progenitor cells following standard Cy/Flu conditioning was shown to mediate ACT failure, and ACT function could be rescued by IL-6 blockade administered the day prior to conditioning Cy/Flu [184]. Future studies will need to demonstrate if the combination of CD40-agonist and IL-6 blockade (e.g., tocilizumab) can synergize toward favorable TME remodeling and immune induction in the preparative context of TCR-T or other ACT approaches.

Together, these data indicate that even where tumors are poorly infiltrated with tumor-specific T cells, such cells can be engineered *ex vivo* to enhance tumor infiltration. Moreover, while the tumor blocks T cell function, *ex vivo* engineering can build resistance into the transferred cells. Finally, where antigen presentation is a limitation, exploiting NK cell therapy or the effects of combination with conventional therapies may improve the visibility of tumors to the immune system [185–187]. At present, adoptive T cell therapies are not well-integrated into the conventional treatment paradigm of HNSCC. These are currently experimental therapies for patients who have failed existing treatments or for whom conventional alternatives are not available. As with other immunotherapies, adoptive transfer may be used to change the immune environment of the tumor in conjunction with chemotherapy, radiation therapy, and surgery in HNSCC (Figures 4B,C). As with all therapies, timing may be key, since chemotherapy and radiation therapy can be unforgiving to T cells. Moreover, biomaterial-based intraoperative T cell or immunotherapy delivery may be an efficient means to regulate the immune environment for subsequent control of logoregional disease [188, 189]. The fact that HNSCC is a disease conventionally treated with multimodality therapy makes it essential that novel immunotherapies are appropriately integrated into current treatment paradigms to convert poor responding patients to cures.

## Conclusions

Almost every category of immunotherapy has proven effective in controlling HNSCC tumors in pre-clinical models. These include innate adjuvants, T cell checkpoint inhibitors, T cell costimulators, adoptively transferred T cells, myeloid regulatory targets, vaccines, and a wide range of alternative immune focused therapies. Despite this, there are only a handful of approved immunotherapies for HNSCC in the clinic. It is normal to see promising pre-clinical therapies fail in clinical trials, but it is always valuable to understand the biology behind the wide difference between successes in pre-clinical models and clinical trials.

Firstly, there is a significant impact of pre-clinical model selection. Some tumor models are more responsive to treatment than others. In part this is explained by immunogenicity [8], where some tumors are so close to the precipice of cure that almost any intervention can result in success. In our experience the CT26 colorectal carcinoma is one such model that is responsive to almost all T cell targeted therapies that we test in combination with radiation therapy. It could be argued that it would be more accurate to report the response of 8 different tumor models, rather than eight genetically identical mice each given the same genetically identical cancer cells. There may well be patients who are also highly responsive and so may be well-matched to therapies that succeed in CT26 tumors, but the diversity in tumor genetics, patient genetics, and patient immune status across a clinical trial are vastly greater than the differences between any two pre-clinical models. The limitation in pre-clinical models should be understood and embraced, since they are just models. However, clinical translation of any agent that has only cured one or two highly responsive pre-clinical models does not seem reasonable. We would argue that translation requires a higher bar. Yet, the clinical development of anti-CTLA4 and anti-PD-1 blocking antibodies for cancer was based on single agent treatment of highly responsive CT26 and MC38 tumors [190]. As single agents these fail in most melanoma models [191–194] and most immunotherapies fail in mice with authentic spontaneous genetically engineered tumors of various origin in the original host [195, 196], yet ipilimumab and nivolumab are each effective therapies for melanoma patients [197, 198]. Clearly, we cannot over-rely on the authenticity of pre-clinical models. Secondly, through experimental design we can increase the likelihood of cures in any given pre-clinical model. For example, mice treated with immunotherapies within the first few days of tumor implantation can be cured by many agents, but these therapies fail if the tumor is first allowed to establish [74]. In part this is due to the vaccine effect of tumor implantation as discussed above [74], but also the fact that mature suppressive environments have not yet developed [9]. At the point that single agent immunotherapies start to fail, there may be a further therapeutic window where combination therapies can be effective. Therefore, to test combination therapies we must select models and timings where single agents fail, otherwise we cannot show a need or benefit for the combination. Model selection and experimental design are a normal part of pre-clinical testing, can dramatically impact the success of any given treatment, and should be carefully interpreted to help understand whether they will be relevant to actual clinical scenarios in patients [199].

Due to the complexity and impracticality of testing multiple agents in patients to evaluate their ability to change the tumor immune environment from a negative to a positive state, we may need to turn to *ex vivo* patient-derived modeling. Simple tumor explant models can provide a rapid readout of patient-specific responses to immunotherapy agents [189–202]. In these systems, the short *ex vivo* response of small fragments of fresh tumor tissue can have predictive power for patient responses to the same therapy [202]. Tools that help us predict how a patient's tumor might respond to a therapy can help us select the optimum treatment for an individual. Repeated sampling may allow us to determine whether their immune environment has shifted into one that predicts better OS with conventional therapy, and therefore could allow us to gradually shift a predicted unresponsive patient into a predicted responsive patient. In this setting window of opportunity trials can provide valuable information on patient specific responses to diverse interventions with the potential to shift their immune status [203, 204]. We would propose that treatments would focus on generating a tumor specific CD4 and CD8 T cell population in the tumor, and may rely on therapies that can bring suboptimal T cells into the tumor to become functional anti-tumor effectors. In this, way we can begin to find responses for HNSCC patients who are currently poorly served by current treatments.

As we have discussed, pre-malignancy represents the first opportunity for intervention to alter the course of disease (Table 1) and this has a clear relevance to OSCC. Preventing progression in high-risk patients will have a major impact by avoiding malignancy; however, we propose that even in patients that progress, it is possible that intervention can alter the immune environment and thus impact the response to conventional therapies. It seems clear that a wide range of current interventions are highly dependent on the pre-existing immune status of the patient. As we have discussed, analysis of the tumor immune environment of patients with advanced cancers has identified T cell populations that recognize tumor-associated antigens and are linked to improved outcomes in patients. Providing these cells represent a logical target for intervention in patients that lack such cells (Table 1), and innovative adoptive transfer approaches are in development for HNSCC. However, the mere presence of T cell infiltrates in tumors is generally insufficient. HNSCC patients with high T cell infiltrates are only cured following treatment, and it is reasonable that providing T cells will be a good first step for poor prognosis patients before proceeding to conventional therapy. While the ideal goal would be an immune panacea, the history of HNSCC therapy has been and remains multimodal therapy. Integrating immunotherapy into HNSCC treatment is a practical reality for the foreseeable future.

**Table 1:** Immunotherapy interventions discussed.

**Preventative/pre-malignant**	**References**
Vaccination	[22, 39–41]
PD1	[34, 35]
EGFR	[43]
**Malignant**	**References**
Radiation therapy	[74]
Checkpoint combinations	[75, 80–82, 94–99, 190–194, 197, 198]
TGF beta	[14, 100]
Myeloid regulators	[166, 172–176, 180–184, 189]
Adoptive transfer: TIL	[102–109, 112–118, 188]
Adoptive transfer: TCR-T	[119–122, 124–128]
Adoptive transfer: CAR-T	[129–137]
Adoptive transfer: NK platform	[164–167]

## Author contributions

TD, MG, RL, and SS were jointly responsible for the preparation, writing, and editing of the manuscript and figures. All authors contributed to the article and approved the submitted version.

## Funding

This work was supported by NCI R01CA182311, NCI R01CA244142, and by the Providence Foundation.

## Conflict of interest

RL receives research funding from Clinigen, Incute, Celldex, and Bristol Myers Squibb. MG receives research funding from Bristol Myers Squibb and VIR Biotechnology. SS receives research funding from IMV Inc. and consulting fees from Stanford Burham Prebys Medical Discover Institute. Funders had no role in the preparation of the manuscript. The remaining author declares that the research was conducted in the absence of any commercial or financial relationships that could be construed as a potential conflict of interest.

## Publisher's note

All claims expressed in this article are solely those of the authors and do not necessarily represent those of their affiliated organizations, or those of the publisher, the editors and the reviewers. Any product that may be evaluated in this article, or claim that may be made by its manufacturer, is not guaranteed or endorsed by the publisher.
